# Jute Fiber-Reinforced Polymer Tube-Confined Sisal Fiber-Reinforced Recycled Aggregate Concrete Waste

**DOI:** 10.3390/polym14061260

**Published:** 2022-03-21

**Authors:** Chang Gao, Qiuni Fu, Liang Huang, Libo Yan, Guangming Gu

**Affiliations:** 1 College of Civil Engineering, Hunan University, Changsha 410082, China; gc_grace0730@hnu.edu.cn; 2 Department of Organic and Wood-Based Construction Materials, Technische Universität Braunschweig, Hopfengarten 20, 38102 Braunschweig, Germany; q.fu@tu-braunschweig.de; 3 Centre for Light and Environmentally-Friendly Structures, Fraunhofer Wilhelm-Klauditz-Institut WKI, Bienroder Weg 54E, 38108 Braunschweig, Germany; 4 Kunming Municipal Engineering Design and Research Institute (Group) Co., Ltd., Kunming 650000, China; guguangming@163.com

**Keywords:** recycled aggregate concrete (RAC), jute FRP (JFRP), sisal fiber reinforcement, confined concrete, compressive behavior, theoretical models

## Abstract

In this study, the compressive performance of sisal fiber-reinforced recycled aggregate concrete (SFRAC) composite, confined with jute fiber-reinforced polymer (JFRP) tube (the structure was termed as JFRP–SFRAC) was assessed. A total of 36 cylindrical specimens were tested under uniaxial compression. Three major experimental variables were investigated: (1) the compressive strength of concrete core (i.e., 25.0 MPa and 32.5 MPa), (2) jute fiber orientation angle with respect to the hoop direction of a JFRP tube (i.e., β = 0°, 30° and 45°), and (3) the reinforcement of sisal fiber (i.e., 0% and 0.3% by mass of cement). This study revealed that the prefabricated JFRP tube resulted in a significant enhancement of the compressive strength and deformation ability of RAC and SFRAC. The enhancements in strength and ultimate strain of the composite columns were more pronounced for concrete with a higher strength. The strength and ultimate strain of JFRP-confined specimens decreased with an increase in fiber orientation angle β from 0° to 45°. The sisal fiber reinforcement effectively improved the integrity of the RAC and reduced the propagation of cracks in RAC. The stress–strain behaviors of JFRP–RAC and JFRP–SFRAC were predicted by the Lam and Teng’s model with the revised ultimate condition equations.

## 1. Introduction

During the last decades, the circular economy and sustainable development have aroused growing global consciousness of green urban construction, waste management and the conservation of natural resources. The global construction industry generates an average of 40% of the total waste each year and occupies large volumes of landfills. It is predicted that up to 3 billion tons of construction and demolition wastes (CDWs) were produced in China before 2020. Some countries such as Japan and Germany recycled up to 80% of CDWs, while many other countries have recycled 20–40% of CDWs according to the statistical results up to the year 2018, e.g., 40% for China, 30% for Canada, 28% for Switzerland and 32% for Thailand [1,2,3].

By far, recycled aggregate concrete (RAC) technology has been considered the most effective measure in the Waste-to-Energy industry. The aggregates within RAC are produced from CDWs after screening and crushing processes. A certain number of studies have declared that natural defects exist in recycled aggregates as particle shape defects and micro-cracks, which lead to the high-water absorption and crushing index of recycled aggregates [1,4,5]. Some consistent research conclusions were reached that the recycled concrete exhibited lower strength than ordinary concrete [4], and recycled aggregates caused more significant shrinkage and water absorption in the resulting concrete [1,6]. It is worthy to note that the adopted recycled aggregates were resulted from broad sources, e.g., demolished concrete [7,8,9], bricks [10,11,12,13,14], and ceramic tile [15], and the uncertainty surrounding the sources of recycle aggregates could cause variability in the resulting concrete. Some existing scholarly works concluded that: (1) the compressive strength of recycled concrete was 37% or more lower than that of ordinary concrete, and the adhered mortar had an adverse influence on the concrete’s strength [16], (2) the replacement ratios of recycled coarse aggregates within 25% or recycled fine aggregates within 30% rarely exhibited inferior effects on the strength and durability of the resulting concrete [17], (3) the recycled concrete failed in shear mode with a larger peak strain, but the ductility of recycled concrete was decreased compared with ordinary concrete [17]. 

Considering the above demerits of RAC, appropriate measures have been undertaken to improve the performance of recycled concrete, such as the application of fiber-reinforced polymers (FRP). The FRP jackets served as an external confinement device and created tri-axial compressive condition to enhance the strength and ductility of confined concrete. Research on FRPs used in recycled concrete confinement have reported considerable improvements in the performance of recycled concrete [18,19]. Teng et al. revealed that the strength and ultimate strain of recycled concrete with sufficient FRP confinement were comparable to confined ordinary concrete [20]. Studies also found that the outer confinement of FRP could weaken the effect of replacement percentages or uncertain sources of recycled aggregates on the quality of the resulting concrete [21,22,23,24]. However, synthetic FRP also has the drawbacks of brittleness, non-degradation, and high cost [25,26]. Alternative natural fibers have emerged with growing attention due to their low cost and environmentally friendly features, e.g., flax fibers and coir fibers [26]. Furthermore, some natural fibers exhibited comparable strength and modulus to those of glass fibers [25]. For instance, flax fiber has been used to replace glass fibers as energy absorbers for automotive engineering [25], strengthening the concrete material [27]. Through the experimental studies of flax FRP-confined, coir fiber-reinforced concrete, Yan et al. [27] found that the fiber orientation of an FFRP tube determined the failure modes of the composite columns, and the naturally interfacial bonded specimens presented the largest confinement effectiveness. Ardanuy et al. declared that the sisal strands are the most common fiber used in cement paste, since the fiber could improve the durability of cellulose cement composites by pozzolanic addition or hornification treatments [28]. Silva et al. reduced the potential aging of sisal fiber-reinforced cement composites by lowering the content of Portland cement and Calcium Hydroxide, and confirmed that the sisal fibers were effective in bridging and arresting the cracks of the composite system [29]. Tara et al. demonstrated that the confinement performance of sisal FRP and JFRP was of comparable magnitude to glass FRP confinement characteristics [30], and the durability on normal water and thermal aging of JFRP behaved similarly to artificial FRP [31]. Our former experimental study on the performance of JFRP tube-confined, sisal fiber-reinforced normal concrete found that the JFRP tube presented a significant confinement effect on the core concrete, and sisal fiber inclusion improved the efficiency of confinement [32].

Research on FRP tube-confined natural aggregate concrete has shown that the fiber orientation has considerable influence on the performance of the confinement system [33,34,35]. Sadeghian et al. [33] stated that the strength, ductility and ruptured plane of carbon FRP-confined concrete strongly depended on the fiber orientation of the FRP tube. Arunothayan et al. [34] adopted 3D concrete printing technology for the ultra-high-strength concrete and investigated the influence of fiber orientation. The study found that a high fiber volume ratio would significantly enhance the fiber alignment parallel to the printing direction. Au et al. [35] concluded that FRP-confined concrete with fiber oriented at the hoop direction presented brittle failures, whilst FRP-confined concrete with fiber oriented at directions except for the hoop direction presented ductile failure attributed to the fiber reorientation mechanism.

In this study, the compressive performance of JFRP-confined sisal fiber-reinforced RAC containing recycled clay brick aggregates (termed as JFRP–SFRAC) was investigated. The combined use of natural fibers from agricultural waste and recycled aggregates from CDWs would be beneficial for the sustainable development of constructional industry and lower carbon emissions. The influence of jute fiber orientation (i.e., fiber oriented at angle β = 0°, 30° and 45° with respect to the hoop direction) was investigated. Besides this, the effects of the compressive strength of internal RAC and the sisal fiber-reinforcement on the axial compressive behaviour of JFRP–SFRAC were studied.

## 2. Experimental Works

### 2.1. Material Properties

#### 2.1.1. Sisal Fiber, RAs and RAC

**Figure 1:**
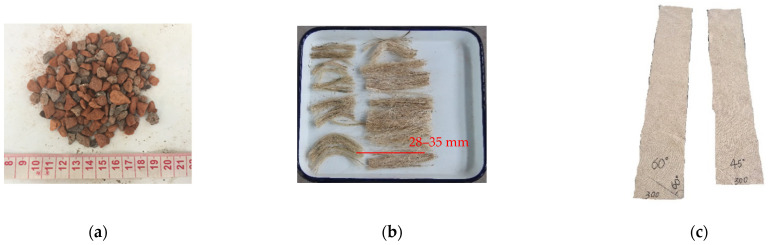
Appearance of constituent materials: (**a**) recycled aggregates with recycled clay brick aggregates, (**b**) sisal fiber and (**c**) jute fabric.

The contents and proportions in RA mixtures are changeable due to the diversity of sources and constitutes of construction and demolition wastes. The RAs used in this study contained about 70% mass content of RAs from clay brick waste and 30% mass content of RAs from concrete waste, as shown in Figure 1a. The constituents and contents of RAs were tested and reported by the manufacture. The physical properties of all types of aggregates were listed in Table 1. The P.O. 42.5R cement was used in the RAC. The physical properties of cement are given in Table 2. The compressive and flexural strength of cement were tested in the study and are given in Table 3.

Based on the earlier studies on JFRP-confined SFPC [32], the SFRAC specimens incorporated sisal fibers weighted at 0.3% of cement mass. The length of the sisal fiber was around 28–35 mm as shown in Figure 1b. The tensile strength, elastic modulus and ultimate strain of the sisal fiber were 363 MPa, 9.0 GPa and 2.2% respectively according to the manufacturer.

Three groups of unconfined RAC and SFRAC cubic specimens with a size of 150 × 150 × 150 mm^3^ were pre-tested under uniaxial compression according to GB/T50081-2002 [36]. Each group (i.e., RAC-a, RAC-b, and SFRAC-b) included six identical cubic specimens and the mix proportions of concrete are presented in Table 3. The average tested compressive strength of category RAC-a, RAC-b and SFRAC-b cubic specimens were 25.0 MPa, 32.5 MPa and 33.5 MPa, respectively. Hence, the mix proportions of RAC in Table 4 were used in the following axial compressive experiments of JFRP–RAC and JFRP–SFRAC.

#### 2.1.2. JFRP Composites

**Figure 2:**
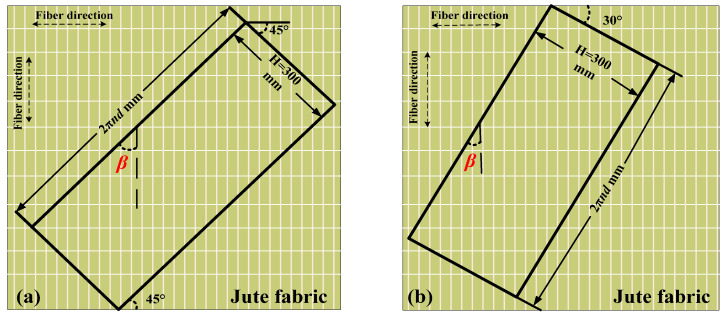
Details of cut jute fabric with different fiber orientations (**a**) β *=* 45°, (**b**) β = 30°.

**Figure 3:**
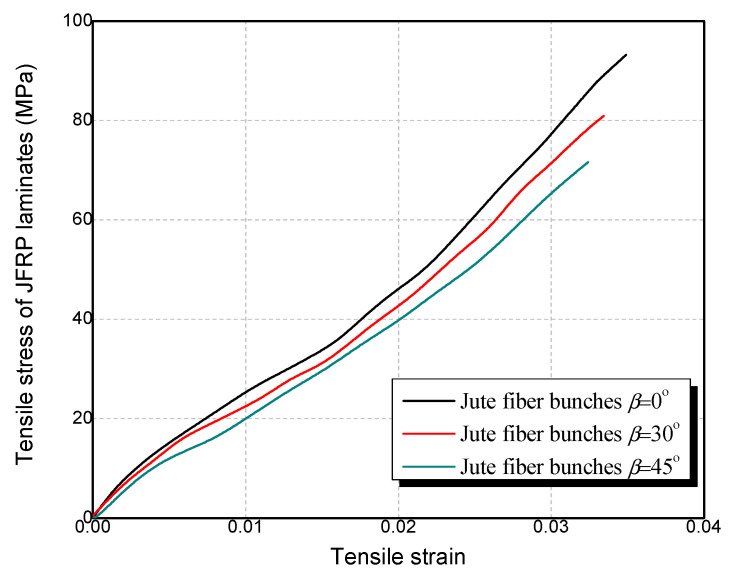
Tensile stress–strain of JFRP laminates.

Bidirectional woven jute fabric with a density of 360 g/m^2^ was used for the fabrication of the JFRP tube as shown in Figure 1c. The JN-C3P epoxy, which consisted of A reagent and B reagent, was used as the matrix of JFRP in this study. The mix proportion of the epoxy was A reagent:B reagent = 5:2. The JFRP laminates were manufactured by dipping the jute fabric into the epoxy and curing at room temperature for 7 days. Tensile tests were conducted on the JFRP laminates to obtain the tensile properties of the JFRP according to ASTM D3039 [37]. The cutting approaches to the jute fabric with 30° and 45° fiber orientation are shown in Figure 2, respectively. The tensile stress–strain behavior of JFRP laminates with different jute fiber orientations is given in Figure 3 and the JFRP composite presented the linear elastic characters. The average tested results and the corresponding standard deviations (SD) of the JFRP laminate tensile tests are listed in Table 5. The tensile strength and strain of JFRP laminates reduced with an increase in the orientation angle β from 0° to 45°. The mechanical properties of different kinds of jute fiber and sisal fiber on the warp direction (i.e., 0°) and weft direction (i.e., 90°) were studied by Codispoti et al. [38], and similar linear-elastic stress–strain behaviors were obtained in their studies. Besides, both the warp direction (i.e., 0°) and weft direction (i.e., 90°) of jute fiber might exhibit similar mechanical properties due to the bidirectional woven characters of jute fabric, and the tensile strength of JFRP laminates would reach their highest when the orientation angle β was 0° or 90° and would reduce when β was in between.

### 2.2. Specimen Preparation

**Figure 4:**
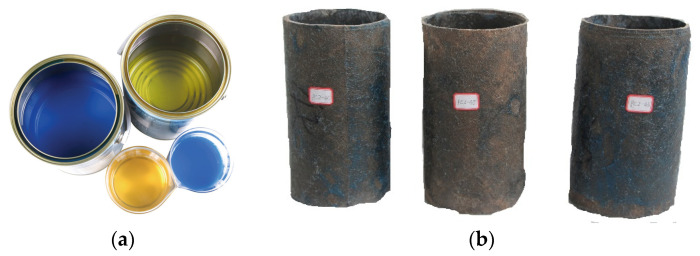
Production of JFRP tubes: (**a**) JN-C3P epoxy, (**b**) prefabricated JFRP tube.

The hand lay-up approach was used for JFRP tube prefabrication and the prefabricated JFRP tubes are shown in Figure 4. Figure 4a shows the A reagent and B reagent of the epoxy. The overlapping length of the JFRP tubes was a quarter of the circumference of the cylinders. The concrete was cast into the JFRP tubes after the tubes were cured for 7 days. All the specimens were maintained in the standard curing room for 28 days. For SFRAC, the sisal fibers were firstly mixed with dry aggregates, considering the agglomeration trend of short sisal fibers. The agglomeration of sisal fibers would lead to nonuniform dispersion and stress concentration in the resulting concrete.

### 2.3. Test Matrix

A total of 36 composite columns (i.e., 18 JFRP–RAC cylinders, 9 JFRP–SFRAC cylinders, 3 unconfined SFRAC and 6 unconfined RAC cylinders) were constructed for the uniaxial compression tests. The size of all specimens was diameter × height = 150 mm × 300 mm. The tested variables included: (1) the inclusion of sisal fiber in RAC (i.e., 0% and 0.3% by mass of cement according to ref. [31]); (2) the compressive strength of the plain RAC (i.e., 25.0 and 32.5 MPa); and (3) the jute fiber orientation of the JFRP tube (i.e., the fiber oriented at β = 0°, 30° and 45°). The 36 specimens in total were divided into 12 groups (i.e., 3 identical specimens were tested for each group) and a code was given for each group, as shown in Table 6. In Table 6, “RAC” denotes the recycled aggregate concrete, “SFRAC” denotes the sisal fiber-reinforced RAC, “JFRP–RAC” denotes the JFRP tube-confined RAC, “JFRP–SFRAC” denotes the JFRP tube-confined SFRAC, “a” and “b” indicate the compressive strength of the inner RAC as 25.0 MPa and 32.5 MPa respectively, and “0”, “30” and “45” denote the jute fiber orientations of 0°, 30° and 45° in the JFRP tube, respectively. For example, the specimen JFRP–SFRAC-b30 represents the specimen of JFRP tube-confined SFRAC, with a jute fiber orientation of 30°, sisal fiber mass content of 0.3% and compressive strength of the internal untreated RAC of 32.5 MPa.

### 2.4. Test Setup

**Figure 5:**
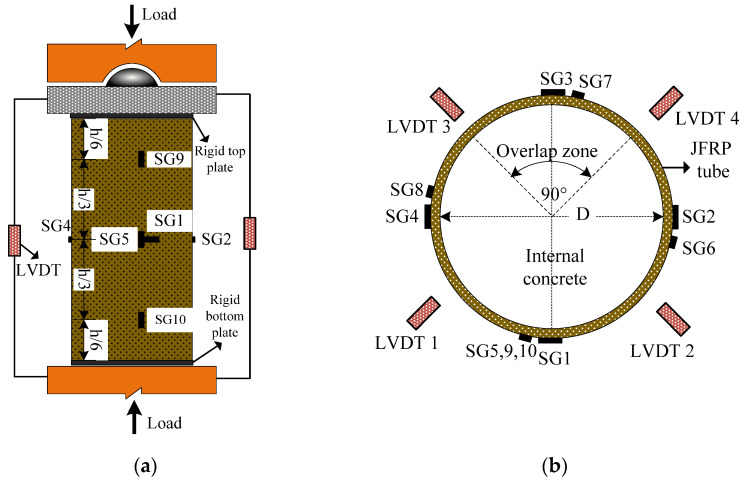
Test setup of the axial compressive tests: (**a**) overview and (**b**) the placement of strain gauges.

Monotonic axial compression on all the specimens was realized by using an MTS testing machine with capacity of 2000 kN as shown in Figure 5a. Rigid steel plates were set on both ends of the cylinders to ensure uniformly distributed loading conditions. The loading procedure was displacement-controlled at a rate of 0.2 mm/min. The longitudinal and hoop strains of the JFRP tube were tested with four evenly distributed straingauges respectively as shown in Figure 5. Considering the influence of the overlap zone, a pair of longitudinal and hoop strain gauges was set in the overlap zone for the potential unconformity of strain in the overlap zone. Two extra longitudinal strain gauges were mounted on both ends of cylinders to measure the longitudinal deformation feature of the entire cylinders (i.e., SG9 and SG10). The longitudinal deformation was also measured by linear variable differential transformers (LVDTs) which were uniformly placed around the cylinders.

## 3. Test Results and Discussion

### 3.1. Failure Modes

**Figure 6:**
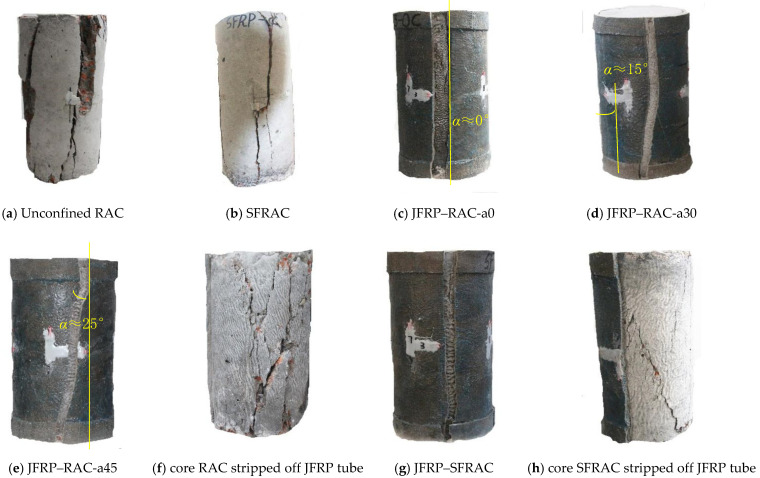
Failure modes of different type of samples.

The failure modes of each kind of specimens are shown in Figure 6. To show the failure modes of the inner concrete in JFRP jackets, the concrete cylinders were stripped off and shown in Figure 6f,h. The cracking patterns and failure mechanisms for JFRP–RAC and JFRP–SFRAC composite columns were compared. The loading process was carried out from the axial stress *σ* = 0 to *σ* = *f_co_* and stopped at *σ* = 0.2*f_co_* in the descending stage. The surface cracks in unconfined RAC appeared until the axial stress reached *σ* > 0.4*f_co_* and developed with cover concrete flaked off. The failure mode of unconfined RAC cylinders exhibited several thorough longitudinal cracks and flaked off bulk concrete rubble, as illustrated in Figure 6a. Compared with unconfined RAC categories, fewer and narrower longitudinal cracks in SFRAC specimens were observed, and the integrity of the cover concrete was maintained better as shown in Figure 6b. For unconfined SFRAC specimens, the serrated cracks were observed instead of linear cracks of unconfined RAC specimens due to the good tensile property of sisal fiber which limited the lateral expansion of concrete.

For the JFRP–RAC specimens, no obvious damage was observed until the axial stress *σ* reached 0.9*f_ct_*, when a tearing sound was heard. All JFRP–RAC specimens ruptured with one single longitudinal thorough crack of the outer JFRP tube with a width of around 1.1–1.3 cm, and the inner concrete was crushed thoroughly with several short longitudinal cracks as shown in Figure 6c–f. Due to the extra JFRP strengthening at the ends of specimens, the core concrete in the middle of the specimens crushed roughly which formed the inverted cone ruptured planes. The angle α between the longitudinal thorough crack of the JFRP tube and the vertical direction was defined to describe the influence of different jute fiber orientations of angle β on the failure modes. For jute fiber oriented at β = 0°, 30° and 45°, the angles α were approximately equal to 0°, 15° and 25°, respectively, as shown in Figure 6c–e. For JFRP–SFRAC with jute fiber oriented at 0°, a similar failure mode was observed in which the outer JFRP tube failed with one single vertical thorough crack, as shown in Figure 6g. The SFRAC core in SFRAC–JFRP specimens ruptured with limited thorough longitudinal cracks and the failed plane tended to be more integrated due to the bridging effect of sisal fiber compared with the RAC core in RAC–JFRP specimens, as shown in Figure 6h. Compared to the study of K. Madhavi et al. [39] on the compressive behavior of concrete cylinders wrapped with jute fiber composites, the jute-polyester-wrapped cylinders experienced cohesive failure by crushing of the concrete. The reason for the different failure modes could be the smaller confinement stiffness of JFRP than that in this paper, or that the confinement effect in JFRP-wrapped concrete is not as significant as that in JFRP tube-encased concrete used in this paper. For the studies on the compressive behavior of concrete filled with flax FRP tube cylinders, e.g., Bai et al. [40], their failure modes were similar to that of JFRP-confined RAC in this paper.

### 3.2. Compressive Stress–Strain Behavior and Ultimate Condition

#### 3.2.1. Axial Stress–Strain Curves

**Figure 7:**
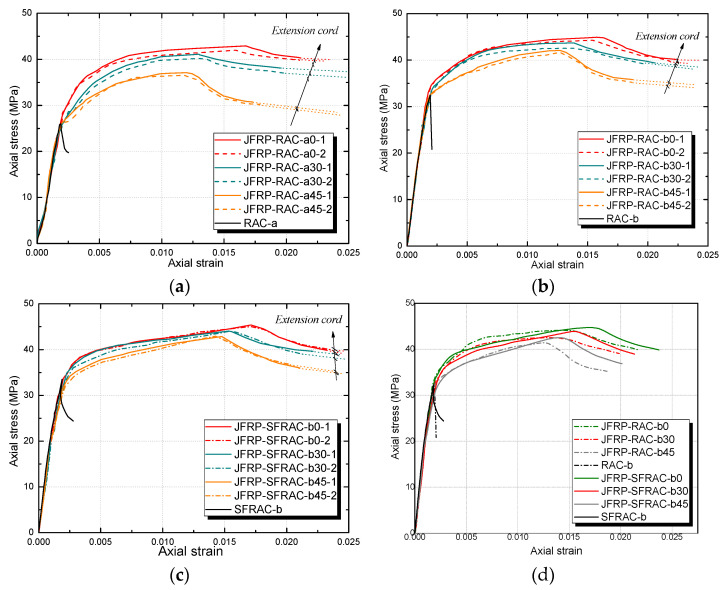
The axial stress–strain relationships of JFRP–RAC and JFRP–SFRAC: (**a**) JFRP–RAC with different orientations of jute fibers (*f_co_* = 25.0 MPa); (**b**) JFRP–RAC with different orientations of jute fibers (*f_co_* = 32.5 MPa); (**c**) JFRP–SFRAC with different orientations of jute fibers (*f_co_* = 33.5 MPa); (**d**) axial stress–strain of JFRP–RAC vs. JFRP–SFRAC.

Figure 7 shows the axial stress–strain responses of JFRP–RAC and JFRP–SFRAC. The JFRP–RAC and JFRP–SFRAC cylinders with different orientations of jute fiber presented similar axial stress–strain curves. The stress–strain responses of both JFRP–RAC and JFRP–SFRAC specimens could be characterized as three stages: the initial linear steep ascending stage, the second placid non-linear ascending stage until the peak stress, and the third non-linear slowly descending stage. The absolute value of the slopes of the third descending stage increased with an increase in angle β of the fiber orientation. For each axial stress–strain curve of the specimens, one extension cord of the final descending stage to predict the trend of the axial stress–strain curves were added, as shown in Figure 7a–c. The descending stages would develop smoothly along the extension cords. Then, the extended stress–strain curves of the JFRP–RAC specimens were compared with those of the JFRP–SFRAC specimens, as shown in Figure 7d. Due to the bridging effect of sisal fiber reinforcement in the core SFRAC, the sisal fiber tended to retard the lateral expansion of core concrete and limit the cracking or crushing of the inner concrete. Both the second stage (i.e., the placid non-linear ascending stage until the peak stress point) and third stage (i.e., the descending stage following the peak stress point) of JFRP–SFRAC specimens were extended compared to those of JFRP–RAC specimens, which exhibited a more significant ductile failing process.

**Figure 8:**
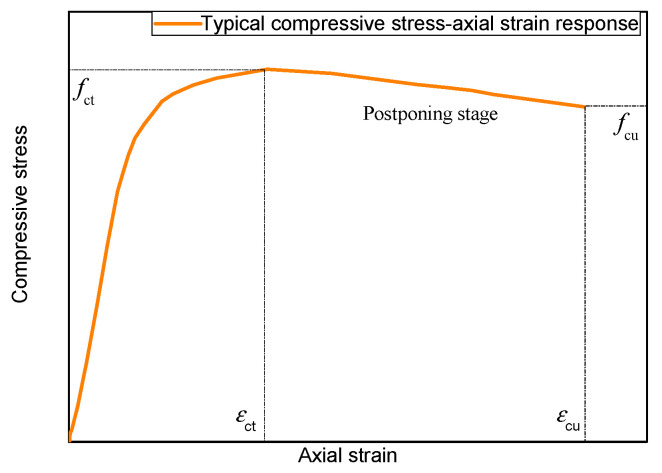
The typical stress–strain relationship of JFRP–RAC and JFRP–SFRAC.

Figure 8 shows the simplified axial stress–strain curves for JFRP–RAC and JFRP–SFRAC specimens. Two key points were drawn to determine the feature of this response, i.e., the top stress point (TP) corresponding to the peak stress (i.e., peak axial compressive stress $f_{ct}$ and corresponding axial strain $\varepsilon_{ct}$) and the ultimate condition point (UP) at the end of the curves (i.e., ultimate axial strain $\varepsilon_{cu}$ and corresponding axial stress $f_{cu}$). The JFRP-confined RAC and SFRAC specimens featured a stress–strain response of weak confinement. The weak confinement is described by a small confinement ratio: $f_{l}/f_{co}\;\leq$ 1, where the lateral confining pressure $f_{l}$ was calculated as $f_{l}=2f_{frp}t_{frp}/d$, the $f_{frp}$ and $t_{frp}$ were the tensile strength and thickness of FRP, respectively, and d is the diameter of cylindrical specimens. The strong confinement is described by a large confinement ratio: $f_{l}/f_{co}\;>$ 1. For strong confinement of FRP-confined concrete, the axial stress–strain curves perform as a bilinear ascending shape, and the compressive strength (TP point) and the ultimate axial strain (UP point) would be reached at the same point that represents the rupture of the confinement outside. For the weak confinement, the axial stress–strain curves generally consist of two ascending stages followed by one descending stage, and the compressive strength would be reached before the rupture of the jacket. The weak confinement could also be sufficient confinement as long as the axial stress at UP point was larger than the compressive strength of unconfined concrete [40]. Thus, based on the criteria of confinement level discussed here, it can be concluded that the confinement of JFRP on the RAC and SFRAC was effective, as shown in Figure 7. The possible reasons for the resulting weak confinement of JFRP might be that: (1) the relatively lower tensile strength and larger ultimate strain of jute fiber induced the post-peak stage of stress–strain curves, (2) the thicker JFRP tube caused the lower expanding rate of the core concrete, (3) the relatively limited improvement of JFRP on the compressive strength of RAC leads to the ductile failing process of the composite columns.

#### 3.2.2. Lateral Strain–Axial Strain Relation

**Figure 9:**
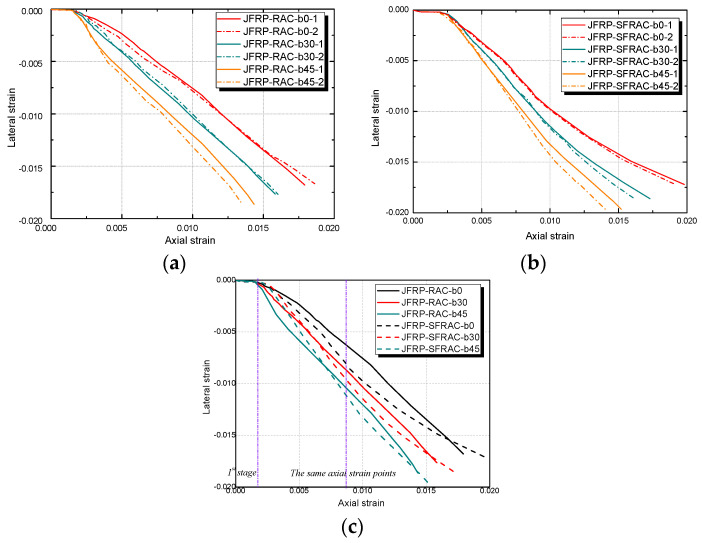
Lateral strain–axial strain of JFRP–RAC and JFRP–SFRAC: (**a**) JFRP–RAC; (**b**) JFRP–SFRAC; (**c**) Comparison between JFRP–RAC and JFRP–SFRAC.

The lateral strain–axial strain relationships of JFRP–RAC and JFRP–SFRAC were given in Figure 9. Both categories of JFRP–RAC and JFRP–SFRAC specimens exhibited similar lateral strain–axial strain $\varepsilon_{l}$–$\varepsilon_{c}$ responses. The lateral–axial strain developed linearly with implicit lateral deformation at the first stage. The confinement of the JFRP tube was not activated and the inner concrete carried most loading at the first stage until reaching the peak strength of the unconfined concrete. At the second stage, the lateral strains increased apparently with the cracking of the inner concrete and expansion of the JFRP tube. By comparing the tested groups of JFRP–RAC and JFRP–SFRAC with different fiber orientation angles of β = 0°, 30° and 45°, the lateral strain–axial strain curves terminated earlier with an increase in β (see Figure 9a,b). This could be attributed to the reduced lateral strains caused by the angle between the fiber orientation and force direction. As shown in Figure 9c, the lateral strains of JFRP–SFRAC were similar to or tended to be smaller than those of JFRP–RAC specimens at the same axial strain points in the initial stage, where the JFRP confinement had not been activated and the lateral expansion was invisible. The later lateral strain of JFRP–SFRAC developed slower than that of the JFRP–RAC specimens. The reason was that the sisal fiber in RAC could restrain the lateral expansion of RAC by maintaining the integrity of inner concrete due to the bridging effect, and by preventing the inner concrete from cracking or crushing.

### 3.3. Compressive Strength and Ultimate Strains

The tested results are summarized in Table 7, where $f_{co}$ and $\varepsilon_{co}$ denote the compressive strength and corresponding ultimate strain of the RAC or SFRAC respectively, $f_{ct}$ and $\varepsilon_{ct}$ denote the peak compressive stress and corresponding axial strain of the JFRP–RAC and JFRP–SFRAC specimens (TP point) respectively, $\varepsilon_{cu}$ and $f_{cu}$ denote the ultimate strain and corresponding stress (UP point) respectively, and $\varepsilon_{h,rup}$ denotes the lateral ruptured strain of JFRP tube at UP point. The strength ratio $\gamma_{f}^{incre}$ was defined as $f_{ct}/f_{co}$ to evaluate the confinement performance of the JFRP tube on the inner concrete, and the ductility index $\gamma_{\varepsilon }^{incre}$ was defined as $\varepsilon_{cu}/\varepsilon_{co}$ to evaluate the ductility and deformation ability of the specimens.

#### 3.3.1. Strength Ratio

The strength enhancement ratio $\gamma_{f}^{incre}$ = $f_{ct}/f_{co}$ was used to quantitatively describe the effect of the composites on the strength improvement of RAC. The condition of 2 $>\gamma_{f}^{incre}>$ 1 indicated that the confinement of the FRP tube on the concrete is effective, and the condition of $\gamma_{f}^{incre}\geq$ 2 indicated the strong confinement of the FRP tube on the concrete. It could be concluded that the confinement of JFRP tube in the study presented effective confinement on RAC and SFRAC. Besides, the condition that the stress of UP point $f_{cu}$ exceeded the strength of unconfinement concrete $f_{co}$ also indicated the effective confinement of JFRP on the RAC and SFRAC in the study [41]. The axial strength enhancement ratios $\gamma_{f}^{incre}$ decreased with an increase in the compressive strength of inner concrete. An increase in the compressive strength of plain RAC lead to limited promotion on the stress both at TP and UP point of JFRP–RAC and JFRP–SFRAC specimens. The increment of compressive strength between unconfined concrete specimens RAC-a ($f_{co}$ = 25 MPa) and RAC-b ($f_{co}$ = 32.5 MPa) was 7.5 MPa, but the increments of compressive strength between JFRP–RAC-a0 and JFRP–RAC-b0 specimens, JFRP–RAC-a30 and JFRP–RAC-b30 specimens, JFRP–RAC-a45 and JFRP–RAC-b45 specimens were 3.0 MPa, 3.4 MPa and 5.5 MPa, respectively. Based on this, the outer JFRP confinement could be considered one effective way to alleviate the inferior influence of the ingredient complexity of recycled aggregates on the mechanical properties and quality of the resulting concrete. The strength ratios $\gamma_{f}^{incre}$ of specimens with different fiber orientations of angle β = 0°, 30° and 45° decreased in sequence. Both the peak stress $f_{ct}$ at TP point and the ultimate stress $f_{cu}$ at the UP point decreased with an increase of the angle β. The admixture of sisal fiber in RAC slightly improved the compressive strength, i.e., the addition of sisal fiber increased the compressive strength from around 0.4 MPa to 1.0 Mpa by comparing the JFRP–RAC-b and JFRP–SFRAC-b specimens.

#### 3.3.2. Ductility Ratio

The ductility ratio $\gamma_{\varepsilon }^{incre}$ was defined as the ratio of ultimate axial strain of confined and unconfined specimens $\varepsilon_{cu}/\varepsilon_{co}$ to evaluate the ductility level of JFRP–RAC and JFRP–SFRAC composite columns. The use of the JFRP tube improved the ductility of the RAC significantly, in that the ductility ratio $\gamma_{\varepsilon }^{incre}$ ranged from 7.18 to 10.62 as shown in Table 5. The JFRP–RAC-b specimens with higher compressive strength of the inner RAC presented larger ductility ratios than those of JFRP–RAC-a specimens. The inclusion of sisal fiber in JFRP–SFRAC-b specimens led to the reduced ductility ratios of the composite columns, such that the sisal fiber reinforcement had effectively limited the propagation of the cracking of RAC. The ductility ratio $\gamma_{\varepsilon }^{incre}$ and ultimate axial strain of JFRP–RAC and JFRP–SFRAC specimens with different fiber orientations decreased with an increase in angle β, while the hoop strain $\varepsilon_{h,rup}$ increased with an increase in angle β. It could be concluded that the JFRP tube with fiber oriented at the hoop direction presented the most significant strength improvement and deformation confinement on the RAC.

## 4. Theoretical Models

### 4.1. Ultimate Condition Equations

Numerous predicted stress–strain models have been developed for traditional FRP-confined circular concrete columns [41,42]. As mentioned in Section 3.2.1, the stress–strain response and the ultimate condition are significantly influenced by the confinement stiffness of FRP and whether sufficient confinement is achieved. The peak axial stress and the ultimate strain equations were given in the literature [41]. Most existing peak axial stress equations were based on the estimated the lateral confining pressure of FRP, $f_{l}$. Most research on FRP-confined concrete has demonstrated that the confinement stiffness of the FRP jacket affects both the strength and the ultimate axial strain of FRP-confined concrete [40]. It is interesting to note that the confinement stiffness of the FRP jacket also affects the type of stress–strain behavior [42]. The early peak axial stress models of Mirmiran and Shahawy [43], Spoelstra and Monti [44], Fam and Rizkalla [45] and Chun and Park [46] used the “five parameters” multiaxial failure surface proposed by Willam and Warnke as Equation (1). Teng et al. proposed equations to define the peak axial stress for sufficiently and insufficiently confined concrete as Equation (2a,b) [47]. The linear function Equation (2a) was employed for sufficiently confined concrete. A minimum value of $f_{l}/f_{co}$ = 0.07 for sufficiently confined concrete was suggested by Spoelstra and Monti [44]. The axial strain model at peak axial stress mostly employed the equation proposed by Richart et al. [48] as Equation (3). Lam and Teng proposed Equation (4a,b) to predict the ultimate axial strain of FRP-confined concrete [47].

**Figure 10:**
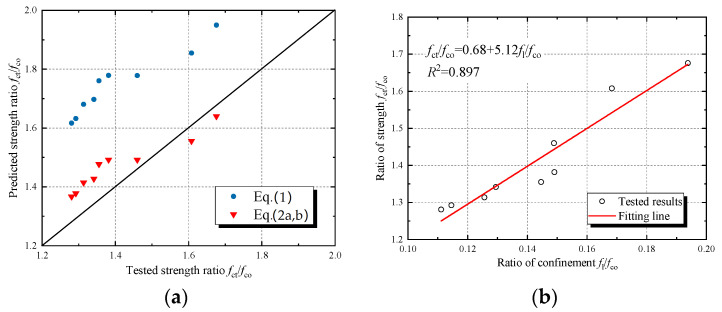
Strength models: (**a**) comparison of predicted strength and tested strength and (**b**) regressed analysis of revised strength model.

**Figure 11:**
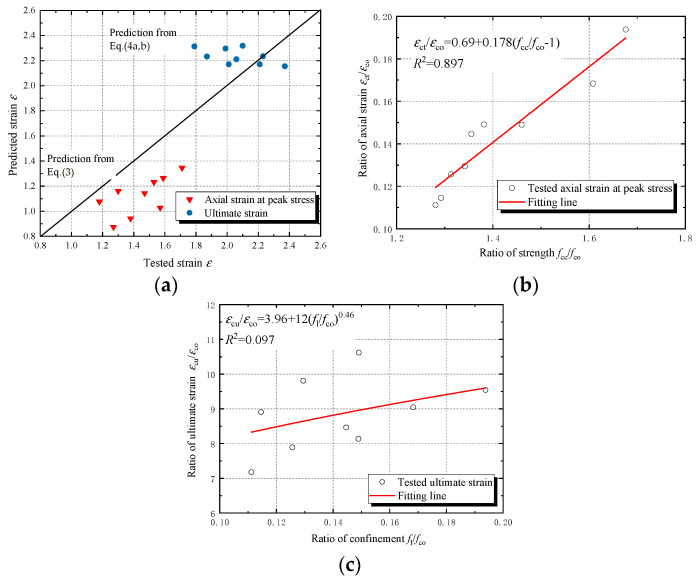
Strain models: (**a**) comparison of predicted strain and tested strain, (**b**) regressed analysis of axial strain model at peak stress and (**c**) regressed analysis of ultimate strain model.

The comparison between the predicted values and the tested values of compressive strength and ultimate strain of JFRP–RAC and JFRP–SFRAC is shown in Figure 10 and Figure 11. Both Equation (1) and Equation (2) overestimated the compressive strength of JFRP–RAC and JFRP–SFRAC. The Equations (3) and (4) were used to predict the axial strain at peak stress and ultimate strain, respectively. The predicted results of axial strain were shown in Figure 11a. The Equation (3) underestimated the axial strain of JFRP–RAC and JFRP–SFRAC at peak stress, and Equation (4) performed relatively well in the prediction of ultimate strain. Due to the deviation of the prediction, the revised strength model and axial strain models were given by regressed analysis of the tested results as Figure 10b and Figure 11b,c, respectively.

(1) $$\frac{f_{ct}}{f_{co}}=2.254\sqrt{1+7.94\frac{f_{l}}{f_{co}}}-2\frac{f_{l}}{f_{co}}-1.254$$

(2a) $$\frac{f_{ct}}{f_{co}}=1+3.3\frac{f_{l}}{f_{co}}\;\;\;\mathrm{for}\;\mathrm{sufficiently}\;\mathrm{confined}\;\mathrm{concrete}$$

(2b) $$\frac{f_{ct}}{f_{co}}=1\;\;\;\;\;\mathrm{for}\;\mathrm{insufficiently}\;\mathrm{confined}\;\mathrm{concrete}$$

(3) $$\varepsilon_{ct}=\varepsilon_{co}\left[1+5\left(\frac{f_{cc}}{f_{co}}-1\right)\right]$$

(4a) $$\frac{\varepsilon_{cu}}{\varepsilon_{co}}=1.75+6.5\rho_{K}^{0.8}\rho_{\varepsilon }^{1.45}$$

(4b) $$\rho_{K}=2E_{f}t_{f}/\left(f_{co}/\varepsilon_{co}\right)d,\;\;\;\rho_{\varepsilon }=\varepsilon_{h,rup}/\varepsilon_{co}$$

### 4.2. Stress–Strain Model

**Figure 12:**
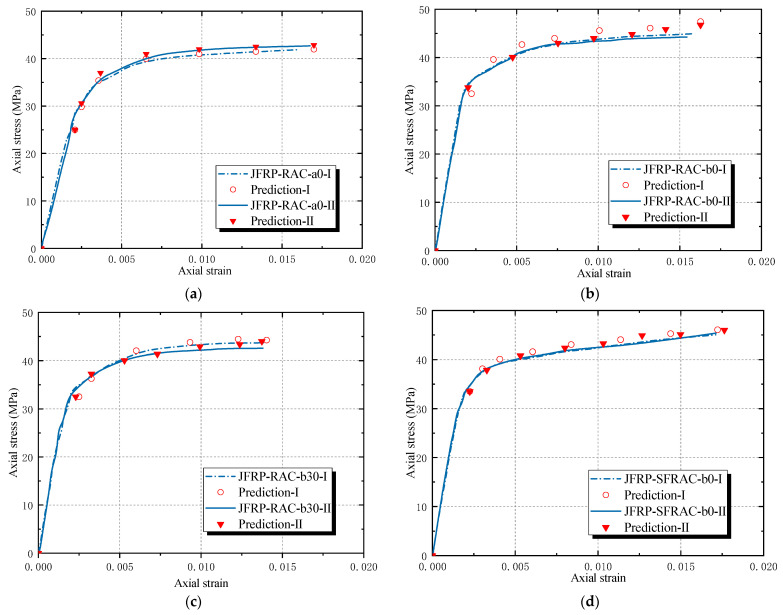
Comparison of predicted and tested stress–strain behavior: (**a**) JFRP–RAC-a0, (**b**) JFRP–RAC-b0, (**c**) JFRP–RAC-b30 and (**d**) JFRP–SFRAC-b0.

Based on the discussion on the ultimate condition equations, Lam and Teng’s stress–strain model was used in the prediction of the stress–strain response of JFRP–RAC and JFRP–SFRAC as Equation (5a,b) [47]. It should be noted that Lam and Teng’s model was based on the assumption that the stress–strain curves consist of an initial parabolic stage and a second linear stage. The predicted axial stress–strain curves of typical specimens JFRP–RAC-a0, JFRP–RAC-b0 and JFRP–RAC-b30 are shown in Figure 12. The trend of predicted axial stress–strain behavior was exhibited as some typical points. The predictions of axial stress–strain behavior were in good agreement with the test results. Based on the revised ultimate condition equations, the stress–strain models performed well for JFRP–RAC and JFRP–SFRAC in the current format.

(5a) $$f_{c}=E_{c}\varepsilon_{c}-\frac{{\left(E_{c}-E_{2}\right)}^{2}}{4f_{co}}\varepsilon_{c}^{2}\;\;\;\;\mathrm{for}\;0\leq \varepsilon_{c}<\varepsilon_{t}$$

(5b) $$f_{c}=f_{co}+E_{2}\varepsilon_{c}\;\;\;\;\mathrm{for}\;\varepsilon_{t}\leq \varepsilon_{c}<\varepsilon_{cu}$$

### 4.3. Lateral-to-Axial Strain Model

**Figure 13:**
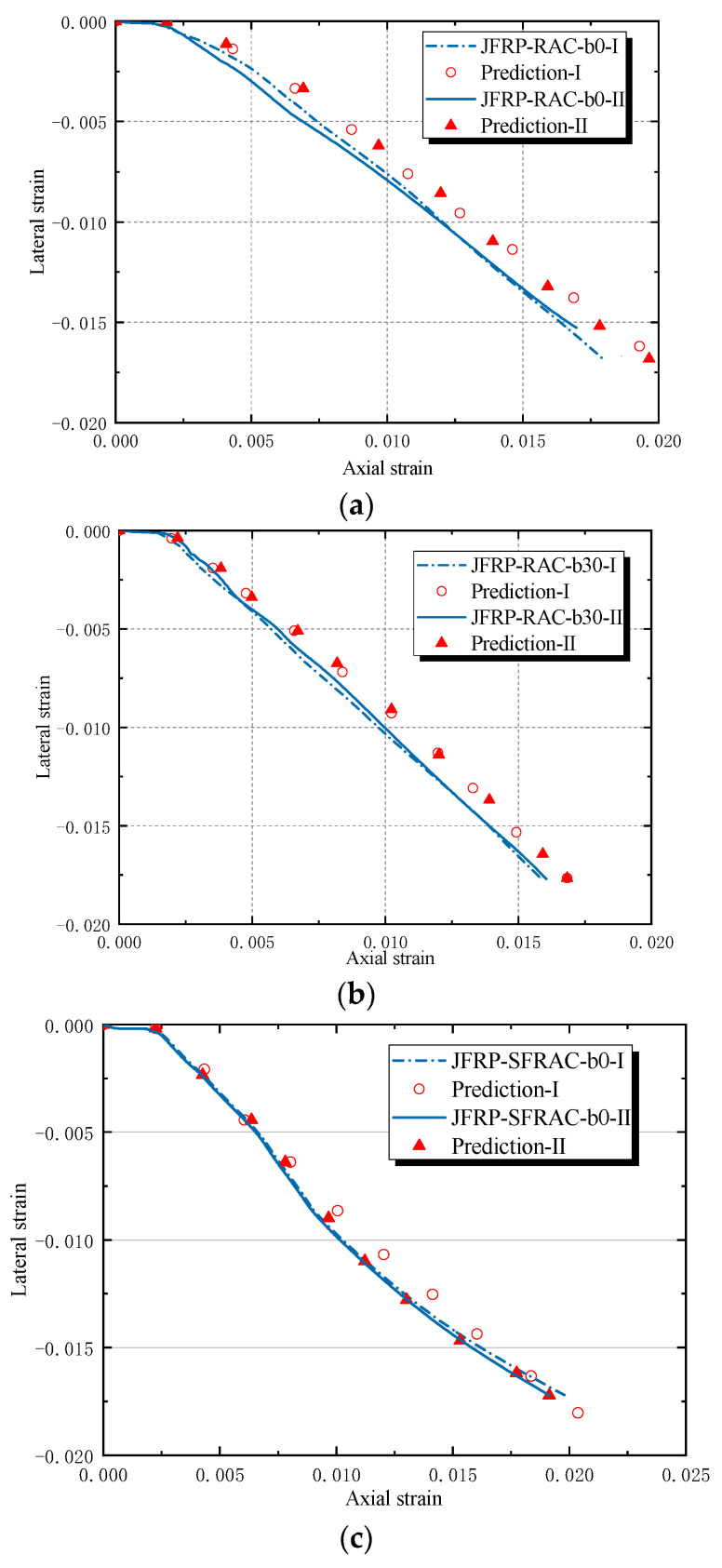
Comparison of Teng’s lateral strain-axial strain model: (**a**) JFRP–RAC-b0, (**b**) JFRP–RAC-b30 and (**c**) JFRP–SFRAC-b0.

The lateral-to-axial strain equation as Equation (6) [49], which is applicable to unconfined, actively confined and FRP confined concrete, was applied in the study and also compared with the tested results. It is evident that Equation (6) of the lateral-to-axial strain model exhibited the two-stage features of lateral-axial strain of JFRP–RAC and JFRP–SFRAC. The predicted lateral-to-axial strain curves consisted of an initial linear stage and a second non-linear stage. The equation slightly overestimated the lateral strain of specimens with the same axial strain as shown in Figure 13.

(6) $$\frac{\varepsilon_{cc}}{\varepsilon_{co}}=0.85\left(1+8\frac{f_{l}}{f_{co}}\right)\left\{{\left[1+0.75\left(\frac{-\varepsilon_{h}}{\varepsilon_{co}}\right)\right]}^{0.7}-\exp\left[-7\left(\frac{-\varepsilon_{h}}{\varepsilon_{co}}\right)\right]\right\}$$

Based on the comparison of the lateral-axial strain response between the predicted values and tested values, the coefficient $k_{sn}$ is applied to revise the Equation (7) by regressed analysis. The coefficient $k_{sn}$ was applied in the lateral-axial strain model and determined as 0.97 by regressed analysis based on the tested data, where $\varepsilon_{h}$ denotes the lateral strain of outer FRP tube.

(7) $$\frac{\varepsilon_{cc}}{\varepsilon_{co}}=\left(0.85+8.85\frac{f_{l}}{f_{co}}\right)k_{sn}\left\{{\left[1+0.75\left(\frac{-\varepsilon_{h}}{\varepsilon_{co}}\right)\right]}^{0.7}-\exp\left[-7\left(\frac{-\varepsilon_{h}}{\varepsilon_{co}}\right)\right]\right\}$$

## 5. Conclusions

In this study, the experimental investigation and theoretical analysis of JFRP tube-confined RAC and SFRAC under axial compression were conducted. Overall, jute FRP confinement and sisal fiber reinforcement have an active effect on the strength and ductility improvement of RAC. The tests and discussion of the reinforcing mechanism and micro-structure on the composite confined RAC cylinders would be further conducted. The following conclusions were drawn:

(1) The confinement of JFRP increased the compressive strength of RAC by 28~68%. The compressive strength of JFRP-confined specimens with 0°, 30° and 45° jute fiber orientations decreased in sequence. The reinforcement of sisal fiber slightly increased the compressive strength of the RAC.
(2) JFRP increased the ultimate strain of RAC and SFRAC by around 6.18 to 9.62 times. The ultimate strain of confined specimens with 0°, 30° and 45° jute fiber orientations decreased slightly in sequence. The sisal fiber reinforcement in RAC increased the ultimate strain of the composite columns.
(3) JFRP confinement was regarded as weak confinement, but the JFRP presented effective confinement because the ultimate strength $f_{cu}$ was larger than the compressive strength of unconfined concrete $f_{co}$.
(4) The sisal fiber reinforcement slowed down the development of the lateral dilation of RAC by reducing the propagation of cracks in the concrete due to the bridging effect.
(5) The ultimate condition equations of FRP confined natural aggregate concrete exhibited deviation in prediction of the ultimate conditions of JFRP–RAC and JFRP–SFRAC. The predicted stress–strain behavior by Lam and Teng’s model with the revised ultimate condition equations demonstrated good agreement with the tested results.
(6) The predicted lateral–axial strain model used in the study presented a slight overestimation of the dilation of JFRP–RAC, and the coefficient $k_{sn}$ was considered to revise the overestimation by regression analysis.

## Figures and Tables

**Table 1:** Physical properties of aggregates used in the tests.

Type of Aggregates	Diameter (mm)	Density (kg/m^3^)	Water Absorption (%)	Moisture Content (%)	Crushing Index (%)
Fine aggregate	0.35–0.50	1480	5.62	3.07	-
Natural coarse aggregates	5–10	1594	4.37	0.29	12.70
Recycled coarse aggregates	≤10	1216	12.00	6.81	50.90

**Table 2:** Physical properties of P.O. 42.5R cement used in the study.

Index	SO_3_	M_g_O	Loss on Ignition	Alkali Content	Fineness	Initial Setting Time	Final Setting Time	Stability
Value	3.06%	1.67%	2.55%	0.57%	80 mm residue: 6.37%	1.82 h	6.43 h	Rex expansion 3.7 mm

**Table 3:** The tested mechanical properties of P.O. 42.5R cement used in the study.

Items	Compressive Strength (MPa)		Flexural Strength (MPa)	
	3 d	28 d	3 d	28 d
Value	23.4	46.7	4.3	6.9

**Table 4:** Mix proportions of RAC and SFRAC.

No.	ω/c	Water (kg/m^3^)	Cement (kg/m^3^)	Fine Natural Aggregate (kg/m^3^)	Coarse Natural Aggregate (kg/m^3^)	Recycled Coarse Aggregate (kg/m^3^)	Replacement Percentage of RAs	Sisal Fiber Content (%)
RAC-a	0.55	260.4	473.5	633.7	403.3	748.9	65%	-
RAC-b	0.40	260.4	651.1	570.7	363.2	674.5	65%	-
SFRAC-b	0.40	260.4	651.1	570.7	363.2	674.5	65%	0.3

**Table 5:** Mechanical properties of JFRP laminates.

Type of FRP	Orientation of Fibers β	Thickness of FRP Laminate *t*_frp_ (mm)	Elastic Modulus (GPa)	Tensile Strength *f*_frp_ (MPa)	SD	Tensile Strain (%)	SD	Price of Fiber Fabric per m^2^ (US)
JFRP ^1^	0°	3.9	2.67	93.16	1.49	3.49	0.17	$0.4
JFRP ^1^	30°	3.9	2.43	80.92	0.88	3.33	0.06	
JFRP ^1^	45°	3.9	2.21	71.61	1.12	3.24	0.03	

^1^ Five replications were tested for each type of FRP.

**Table 6:** Test matrix of cylindrical specimens in this study.

Sample	Number of JFRP Layers	Orientation of Jute Fibers β (°)	Untreated RAC Strength (MPa)	Sisal Fiber Mass Content
RAC-a	-	-	25.0	-
JFRP–RAC-a0	6	0	25.0	-
JFRP–RAC-a30	6	30	25.0	-
JFRP–RAC-a45	6	45	25.0	-
RAC-b	-	-	32.5	-
JFRP–RAC-b0	6	0	32.5	-
JFRP–RAC-b30	6	30	32.5	-
JFRP–RAC-b45	6	45	32.5	-
SFRAC-b	-	-	33.5	0.3%
JFRP–SFRAC-b0	6	0	33.5	0.3%
JFRP–SFRAC-b30	6	30	33.5	0.3%
JFRP–SFRAC-b45	6	45	33.5	0.3%

**Table 7:** The compressive properties of the specimens.

Specimen Group	*f_co_* (MPa)	*ε_co_* (%)	*f_ct_* (MPa)	SD	$\gamma_{f}^{incre}$	*ε_ct_* (%)	*f_cu_* (MPa)	*f_cu_*/*f*_co_	*ε_cu_* (%)	$\gamma_{\varepsilon }^{incre}$	*ε_h,rup_* (%)
JFRP–RAC-a0	25.0	0.22	41.9	1.7	1.67	1.59	39.8	1.59	2.10	9.55	1.66
JFRP–RAC-a30	25.0	0.22	40.2	1.6	1.61	1.30	36.9	1.47	1.99	9.05	1.70
JFRP–RAC-a45	25.0	0.22	36.5	1.6	1.46	1.18	30.2	1.21	1.79	8.14	1.83
JFRP–RAC-b0	32.5	0.21	44.9	1.7	1.38	1.57	40.2	1.24	2.23	10.62	1.68
JFRP–RAC-b30	32.5	0.21	43.6	1.7	1.34	1.38	39.4	1.22	2.06	9.81	1.71
JFRP–RAC-b45	32.5	0.21	42.0	1.7	1.29	1.27	35.8	1.10	1.87	8.91	1.86
JFRP–SFRAC-b0	33.5	0.28	45.4	2.0	1.36	1.71	39.8	1.19	2.37	8.46	1.72
JFRP–SFRAC-b30	33.5	0.28	44.0	2.1	1.31	1.53	39.6	1.18	2.21	7.89	1.86
JFRP–SFRAC-b45	33.5	0.28	42.9	1.6	1.28	1.47	35.9	1.07	2.01	7.18	1.96

## Data Availability

Not applicable.
